# *C. elegans* possess a general program to enter cryptobiosis that allows dauer larvae to survive different kinds of abiotic stress

**DOI:** 10.1038/s41598-020-70311-8

**Published:** 2020-08-10

**Authors:** Vamshidhar R. Gade, Sofia Traikov, Jana Oertel, Karim Fahmy, Teymuras V. Kurzchalia

**Affiliations:** 1 Max Planck Institute of Molecular Cell Biology and Genetics, Pfotenhauerstrasse 108, 01307 Dresden, Germany; 2 Institute of Resource Ecology at the Helmholtz-Zentrum Dresden-Rossendorf, Dresden, Germany

**Keywords:** Biochemistry, Genetics, Physiology

## Abstract

All organisms encounter abiotic stress but only certain organisms are able to cope with extreme conditions and enter into cryptobiosis (hidden life). Previously, we have shown that *C. elegans* dauer larvae can survive severe desiccation (anhydrobiosis), a specific form of cryptobiosis. Entry into anhydrobiosis is preceded by activation of a set of biochemical pathways by exposure to mild desiccation. This process called preconditioning induces elevation of trehalose, intrinsically disordered proteins, polyamines and some other pathways that allow the preservation of cellular functionality in the absence of water. Here, we demonstrate that another stress factor, high osmolarity, activates similar biochemical pathways. The larvae that acquired resistance to high osmotic pressure can also withstand desiccation. In addition, high osmolarity significantly increases the biosynthesis of glycerol making larva tolerant to freezing. Thus, to survive abiotic stress, *C. elegans* activates a combination of genetic and biochemical pathways that serve as a general survival program.

## Introduction

Organisms in nature encounter abiotic stress, defined as negative impact on living organisms of non-living factors (this does not include starvation), but only a few can survive conditions such as complete absence of water or oxygen, high temperature, freezing or extreme salinity. To achieve this, such organisms enter into a state known as anabiosis or cryptobiosis (hidden life), in which they reduce metabolism to an undetectable level^1^. Once conditions become favorable, they exit the cryptobiotic state and resume their reproductive life cycle. There are many spectacular examples where organisms enter a cryptobiotic state, among them are bacterial spores^2^, plant seeds^3^ and multicellular eukaryotes^4–6^. Recently, it was discovered that nematodes from the Siberian permafrost could be revived to life after dwelling up to 45,000 years in ice^7^. Organisms capable of entering cryptobiosis display dramatic differences to living creatures (absence of motility, metabolism and reproduction) but they are still alive, indicating they undergo a peculiar change in their cellular organization. Understanding the mechanisms by which the cryptobiotic state is induced and maintained could provide important insights on fundamental concepts of dead and alive.

Anhydrobiosis, the most common form of cryptobiosis, is observed in both prokaryotes^8^ and eukaryotes^9–16^. Here, organisms can lose up to 95% of their body water and suspend their metabolism. Previously, we demonstrated that dauer larvae of *Caenorhabditis elegans* is a true anhydrobiote and survives to harsh desiccation^17^. These larvae, which are formed in response to unfavorable environmental conditions such as scarcity of food or high population density, have a different metabolic signature than L3 larvae. This difference is manifested in reduced oxygen consumption, heat production and transition to gluconeogenic mode^18^.

To survive harsh desiccation dauer larvae need to be prepared (preconditioned) by exposure to mild desiccation^17^. Preconditioning, is a specific program that comprises extensive remodeling of transcriptome and proteome, affecting the activity of many regulatory and metabolic pathways^19^. Among these are elevation of a disaccharide trehalose, polyamine biosynthesis, fatty acid desaturation pathways and massive upregulation of an intrinsically disordered protein, LEA-1. Recently it was shown that similar to *C. elegans* dauer larvae, tardigrades upon preconditioning survive to extreme desiccation^20^. Preconditioning upregulates several intrinsically disordered proteins (IDP) essential for desiccation tolerance. These IDPs might protect them against severe desiccation by forming glass-like amorphous matrix^21^. Recent studies have demonstrated that IDPs mediate stress responses by their ability to undergo liquid–liquid phase separation (LLPS)^22,23^.

It remains unclear, whether these pathways are induced only by mild desiccation or other stress factors can induce them as well. Here, we show that elevated osmotic pressure induces similar pathways to preconditioning with mild desiccation and makes them resistant to harsh desiccation. Remarkably, in addition to previously described pathways, high osmotic pressure induces also upregulation of glycerol that enables dauer larvae to survive freezing. Our data indicate that dauer larvae possess a general program to survive different kinds of abiotic stress.

## Results

### Osmotic preconditioning enhances survival of *C. elegans* dauer larvae subjected to harsh desiccation

**Figure 1:**
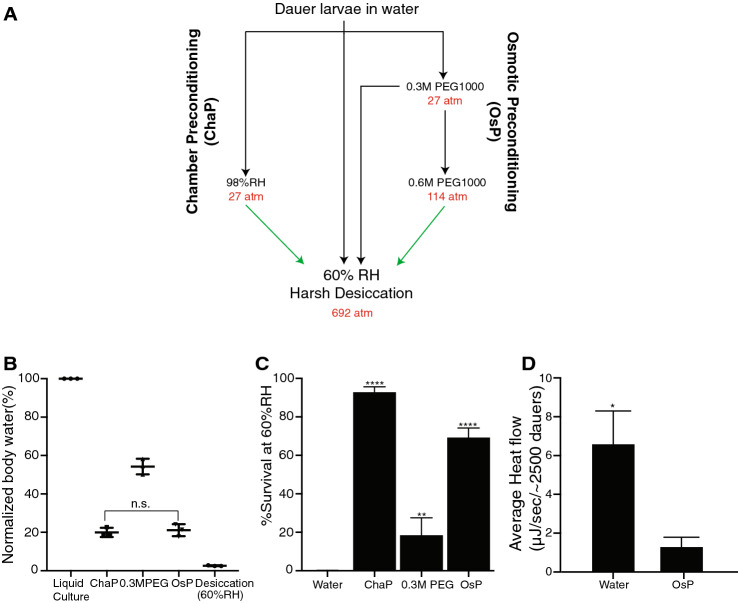
Osmotic preconditioning renders *C. elegans* dauers resistant to harsh desiccation. (**A**) Schematic diagram illustrating exposure of osmotic and chamber preconditioned dauers to harsh desiccation. (**B**) Osmotic and chamber preconditioned *daf-2*(*e1370*) dauers lose similar amounts of water. Error bars indicate standard deviation of three biological replicates. Unpaired two-tailed *t* test with Welch correction, n.s. p > 0.05. (**C**) Osmotic preconditioning enhances survival of *daf-2*(*e1370*) dauers to harsh desiccation. For water n = 2,758, ChaP n = 2,588, 0.3 M PEG1000 n = 2,488, OsP n = 4,113. Error bars indicate standard deviation of three independent experiments with two technical replicates. Statistical comparison was performed with one-way ANOVA with Dunnett’s multiple comparisons test. **p < 0.01, ****p < 0.0001. (**D**) Osmotic preconditioned *daf-2*(*e1370*) dauers have reduced heat flow (heat dissipation per unit time) in contrast to non-preconditioned dauers. Error bars indicate standard deviation of three biological replicates with four technical replicates performed on two different days. Statistical comparison was performed with unpaired two-tailed *t* test with Welch correction. *p < 0.05.

As mentioned earlier *C. elegans* dauer larva survives to harsh desiccation only when the larvae are preconditioned at mild desiccation (98% RH) for 4 days^17^. Here in, this preconditioning will be defined as chamber preconditioning (ChaP). We asked whether other stress factors like high osmolarity, can precondition the dauer larva to survive harsh desiccation. To obtain uniform dauer population of same age, we used dauer constitutive strain *daf-2*(*e1370*) that forms 100% dauer larvae at 25 °C^24^*.* As a starting point of our efforts to develop osmotic preconditioning (OsP), we decided to treat dauer larvae with osmotic pressure comparable to ChaP. Osmotic pressure of air at a given relative humidity was calculated using modified van’t Hoff’s equation and corresponds to 27 atm for 98% RH (Fig. 1A)^17^. Under these conditions, larvae lose about 80% of their body water (Fig. 1B). For OsP we applied the membrane impermeable polyethylene glycol (PEG1000). Using van’t Hoff’s equation and published experimental data, we determined that an osmotic pressure of 27 atm is achieved by 0.3 M PEG^25,26^. However, the water loss at this concentration of PEG is much lower than with ChaP (about 40%, Fig. 1B). The dauer larvae exposed to this concentration of PEG were sensitive to harsh desiccation (Fig. 1C), although the treatment induced high levels of trehalose (Supplementary Fig. S1A) which is essential for desiccation tolerance. We reasoned that in addition to elevated trehalose, in order to be effective, a preconditioning procedure should deplete more water. This was achieved by subsequent exposure to 0.6 M PEG. Thus, osmotic preconditioning (OsP) (Fig. 1A) can be achieved using a two-step treatment with PEG. It should be mentioned that in our preliminary experiments, the larvae directly exposed to 0.6 M PEG showed negligible survival and thus in the following only two-step procedure was used. The amount of water loss in dauer larvae upon OsP was indeed similar to amounts in ChaP treated dauer larvae (Fig. 1B) and furthermore the survival in response to harsh desiccation was significantly increased (Fig. 1C). Resistance to desiccation via osmotic preconditioning is not only specific to *daf-2*(*1370*) dauer larvae but also to wild type (N2) dauer larvae (Supplementary Fig. S4A, B).

Cryptobiosis is associated with cessation of the metabolism. Measurement of the true caloric output of an organism during cryptobiosis is difficult^27^. Calorimetry of a dauer larvae experiencing ChaP or harsh desiccation is not possible due to inability to maintain a constant relative humidity during the measurement. In particular, calorimetric vials are not equipped to contain hygroscopic medium such as sodium hydroxide or calcium chloride, thus constant relative humidity cannot be maintained due to change in vapor equilibrium^28^. OsP occurs in a fluid medium and therefore provides an opportunity to estimate the metabolic rate of preconditioned dauer larvae. As a proxy of metabolic rate, heat flow was used^29^ to estimate the average metabolic rate of OsP-treated dauer larvae. Heat flow was significantly decreased by OsP treatment compared to non-preconditioned larvae (approximately 16% of non-preconditioned larvae, Fig. 1D). These results indicate that dauer larvae subjected to OsP reduces metabolism to low levels.

### Osmotic preconditioning and chamber preconditioning induce similar biochemical pathways

**Figure 2:**
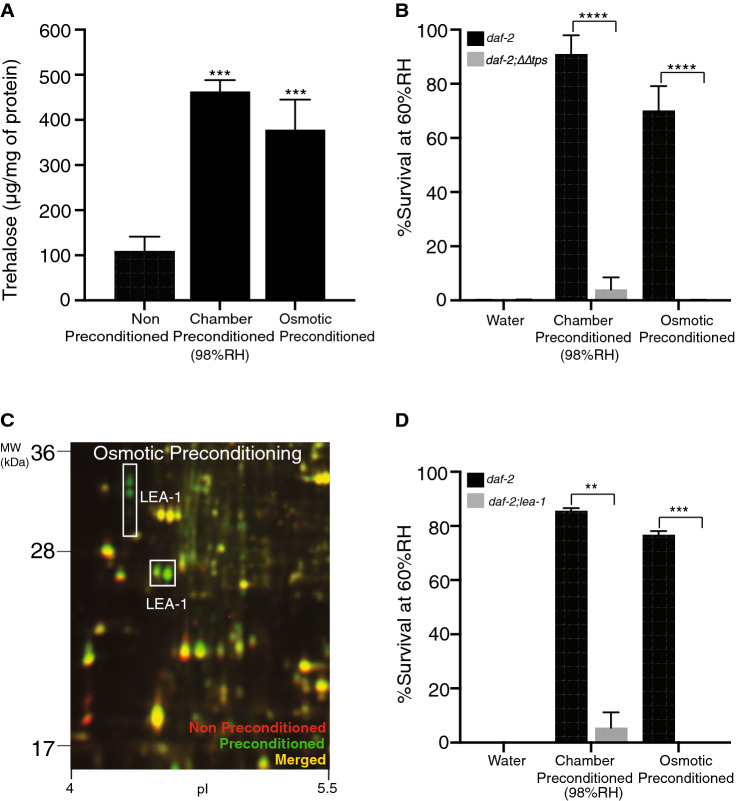
Trehalose and LEA-1 are upregulated upon osmotic preconditioning in *C. elegans* dauers. (**A**) Osmotic preconditioning induces elevation of trehalose levels in dauers. Error bars indicate standard deviation of three independent experiments with three technical replicates. Statistical comparison was performed with one-way ANOVA with Dunnett’s multiple comparisons test. ****p < 0.0001. (**B**) Trehalose elevation upon osmotic preconditioning is essential for desiccation tolerance in dauers. Survival rates of ChaP and OsP *daf-2*(*e1370*),* daf-2;ΔΔ tps* dauer larvae to harsh desiccation. For *daf-2*(*e1370*) water n = 1,912, ChaP n = 2,027, OsP n = 4,334, *daf-2;ΔΔ tps* water n = 1,017, ChaP n = 1,401, OsP n = 1,839. Error bars indicate standard deviation of two independent experiments with two technical replicates. Statistical comparison was performed with unpaired t-test using Holm–Sidak method. ****p < 0.0001. (**C**) Comparison of proteomic changes in *daf-2*(*e1370*) dauers upon osmotic preconditioning. Overlay of false-colored 2D-DIGE images comparing dauer proteomes before (red) and after (green) osmotic preconditioning. Representative portion of the proteome is shown. (**D**) LEA-1 upregulation upon osmotic preconditioning is essential for desiccation tolerance. Survival rates of ChaP and OsP *daf-2*(*e1370*), *daf-2; lea-1*(*tag1676*) dauer larvae to harsh desiccation. For *daf-2*(*e1370*) water n = 1,192, ChaP n = 1,581, OsP n = 5,347, *daf-2; lea-1*(*tag1676*) water n = 521, ChaP n = 1,060, OsP n = 1,448. Error bars indicate standard deviation of two independent experiments with two technical replicates. Statistical comparison was performed with unpaired *t* test using Holm–Sidak method. **p < 0.01, ***p < 0.001. All the experiments were performed in the *daf-2*(*e1370*) background unless otherwise stated.

ChaP induces several biochemical pathways that are essential for the desiccation tolerance, including elevated biosynthesis of trehalose and an intrinsically disordered protein LEA-1^19^. We therefore asked whether OsP also affects these pathways. As shown in Fig. 2A, OsP of larvae induces up to 3.4-fold enhancement of trehalose levels. Moreover, similar to ChaP, the mutants deficient in trehalose biosynthesis pathway (*daf-2;ΔΔtps*)^17^ do not survive desiccation when preconditioned by OsP (Fig. 2B).

Several isoforms of LEA-1 were strongly expressed upon OsP treatment (Fig. 2C).

Previously, we reported that *lea-1*(*RNAi*) renders dauer larvae very sensitive to desiccation^19^. Similarly, a mutant bearing a complete deletion of *lea-1* displayed very poor survival upon desiccation following both ChaP and OsP (Fig. 2D). These results demonstrate that similar to ChaP, OsP induces elevation of trehalose biosynthesis and LEA-1 in dauer larvae and that these are essential for survival under harsh desiccation. Thus, similar pathways seem to mediate the effect of different preconditioning protocols on survival following desiccation.

### In addition to trehalose elevation, osmotic preconditioning induces glycerol biosynthesis

**Figure 3:**
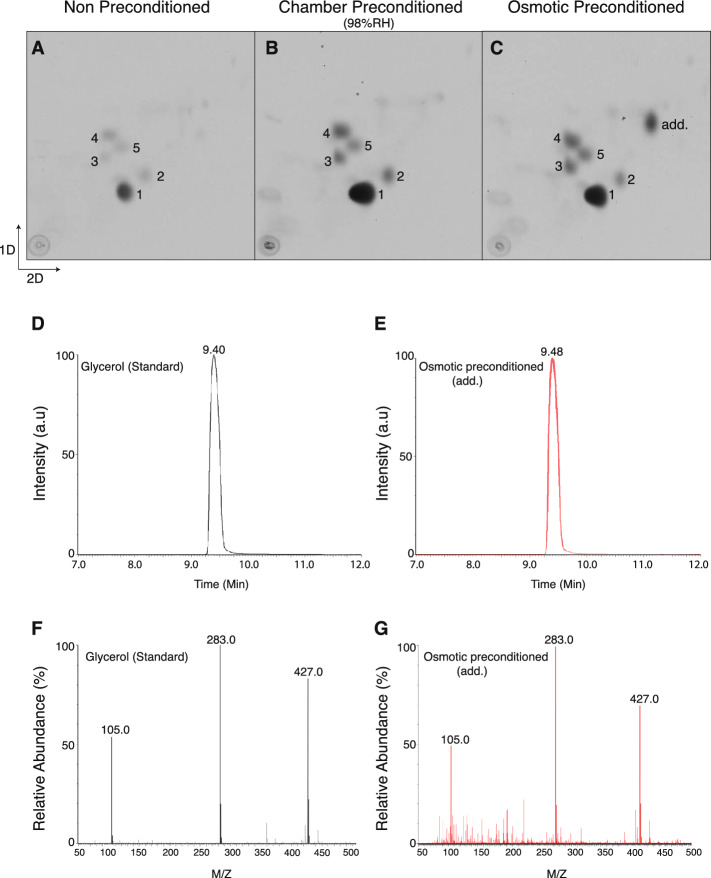
Osmotic preconditioning induces elevation of glycerol biosynthesis. (**A**–**C**) 2D-thin layer chromatography of ^14^C-acetate labelled metabolites from dauers that were non-preconditioned, chamber and osmotic preconditioned respectively. Enumerated spots indicate trehalose (1), glucose (2), glutamate (3), glutamine (4), and alanine (5). Representative images from at least two independent experiments performed on two different days. (**D**,**E**) Chromatogram of glycerol standard and spot add. scrapped out from osmotic preconditioned 2D-TLC. (**F**,**G**) Mass spectrum of glycerol standard and spot add. from osmotic preconditioned sample.

**Figure 4:**
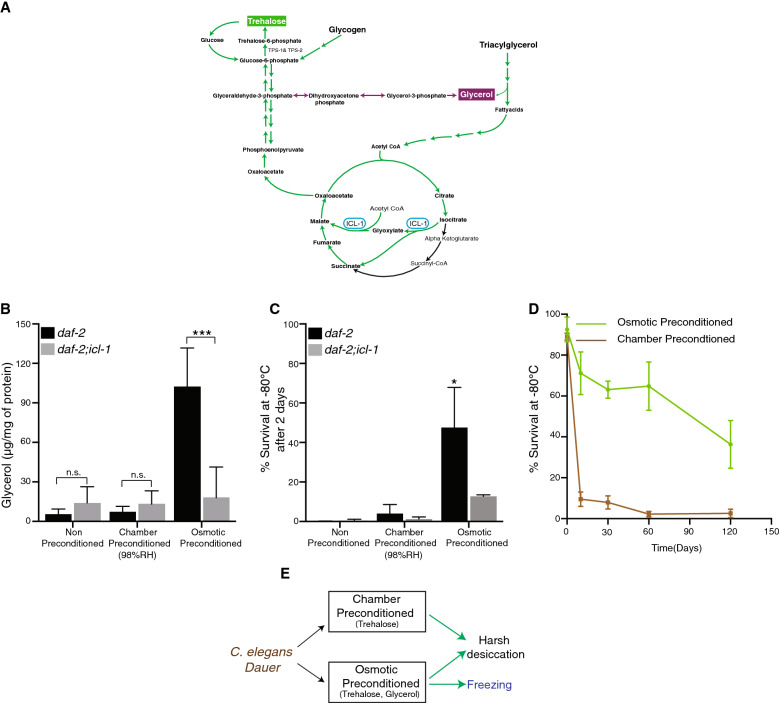
Glycerol upregulation upon osmotic preconditioning is essential for freezing tolerance. (**A**) Schematic diagram illustrating the gluconeogenic mode of dauer larvae. (**B**) Dauer larvae utilize glyoxylate shunt for elevation of glycerol upon osmotic preconditioning. Error bars indicate standard deviation from three independent experiments with three technical replicates. Statistical comparison was performed with unpaired *t* test with Welch correction. n.s. p > 0.05, *p < 0.05. (**C**) Glyoxylate shunt is essential for osmotic preconditioned dauer larvae to survive freezing. Survival rates of *daf-2*(*1370*),* daf-2; icl-1*(*ok 531*) dauer larvae to freezing. For *daf-2*(*e1370*) water n = 783, ChaP n = 1,331, OsP n = 1,802, *daf-2; icl-1*(*ok 531*) water n = 937, ChaP n = 2,082, OsP n = 2,684. Error bars indicate standard deviation of two biological replicates with two technical replicates. Statistical comparison was performed with two-way ANOVA with Sidak’s multiple comparisons test. *p < 0.05. (**D**) Osmotic preconditioned *daf-2*(*e1370*) dauer larvae survive to freezing for longer periods. For OsP n = 17,305, ChaP n = 7,763. Error bars indicate standard error of mean of three biological replicates with two technical replicates. (**E**) Schematic diagram illustrating that osmotic preconditioning enhances survival of *C. elegans* dauer larvae to desiccation and freezing.

We previously showed that a major source of elevated trehalose during ChaP is acetyl-CoA, which is derived from the beta-oxidation of fatty acids^18^. Acetyl-CoA is diverted from the TCA (tricarboxylic acid cycle) via glyoxylate shunt and is used for gluconeogenesis, which culminates in trehalose synthesis (Fig. 4A). We therefore asked whether OsP treated dauer larvae display a similar metabolic trait. To address this question, we radioactively labelled metabolites derived from ^14^C-acetate. As shown by 2D-high performance thin layer chromatography (HPTLC), the most abundant radioactive labelled metabolite extracted was trehalose (Fig. 3A). Smaller amounts of glucose, glutamate, glutamine and alanine were also observed (Fig. 3A). ChaP increased trehalose levels (Fig. 3B) drastically, and the levels of glutamate, glutamine and alanine were also elevated, as reported previously^18^. OsP induced similar changes (Fig. 3C), although we observed an additional spot (add.) that was undetectable in non-preconditioned or under ChaP (Fig. 3C, add.) dauer larvae. This spot was also detected in TLC of non-radioactive samples upon OsP but not ChaP, suggesting that the corresponding compound is produced in large amounts (Supplementary Fig. S2C). This spot had similar mobility to glycerol on 2D-HPTLC (Supplementary Fig. S2C). We further resolved the spot on HPLC (Fig. 3D,E) and using mass spectrometry we confirmed the identity of this spot as glycerol (Fig. 3F,G).

### Osmotic preconditioning induced glycerol is essential for freezing tolerance

Measurement of glycerol levels during both types of preconditioning confirmed that OsP leads to an approximate 15-fold increase in glycerol compared to ChaP (Fig. 4B). Additionally, OsP upregulated glycerol levels in wild type (N2) dauer larvae (Supplementary Fig. S4D), indicating that the elevation is independent of *daf-2*(*e1370*) background. Since trehalose is derived from fatty acids via glyoxylate shunt (Fig. 4A), we asked whether the elevation of glycerol also depends on this pathway. To address this question, we quantified glycerol levels in dauer larvae from an *icl-1*(*ok531*) deletion mutant, which lacks a functional isocitrate lyase (ICL-1). This deletion leads to a frame-shit mutation that results in a non-functional glyoxylate shunt. As shown in Fig. 4B, compared to *daf-2*(*e1370*) dauers, *daf-2;icl-1* dauers have significantly reduced amounts of glycerol. These results indicate that glycerol is also derived from acetyl-CoA, and that a functional glyoxylate shunt is instrumental in elevation of not only of trehalose and further survival at harsh desiccation (Supplementary Fig. S3A, S3B), but also for glycerol upon osmotic preconditioning.

Glycerol is widely used as a cryoprotectant in cells or organs^30^. We asked whether OsP induced glycerol elevation brings a survival advantage for dauer larvae upon freezing. Indeed, OsP treated dauer larvae when exposed directly to -80 °C for a brief period (2 days) survive significantly higher than non-preconditioned or ChaP dauer larvae (Fig. 4C). OsP treated wild type (N2) dauer larvae displayed a similar survival rate (Supplementary Fig. S4C). Contrarily, *daf-2;icl-1* dauer larvae, which do not elevate glycerol upon OsP, displayed a significantly decreased survival rate upon freezing (Fig. 4C). We further asked whether OsP treated dauer larvae can survive to freezing for longer periods. As shown in Fig. 4D, a significant proportion of OsP treated larvae survive to freezing for longer periods up to 120 days in contrast to ChaP larvae. Taken together, these results suggest that metabolic adaptation during preconditioning can promote survival of dauer larvae under different abiotic stress conditions.

## Discussion

Extreme environmental conditions present a constant threat to living organisms. In order to survive such extreme conditions, organisms need to have a general stress response program that activates similar pathways. It will be disadvantageous for them to activate specific pathways for withstanding each kind of stress they encounter. This would be demanding from aspects of energy consumption as well as of regulating the response. Thus, comprising a common survival program to resist various kinds of stress could provide an organism with significant advantage. In our work, we demonstrate that *C. elegans* dauer larvae utilize a combination of genetic and biochemical pathways that serves as a general program to enter into cryptobiosis. In particular, different stress factors such as decreased relative humidity or elevated osmolarity (Fig. 4E), can induce similar mechanisms (e.g. biosynthesis of trehalose, various isoforms of LEA-1) that enables the survival under diverse extreme conditions (desiccation, high osmolarity and freezing). We previously reported that TPS-1 and TPS-2 enzymes that catalyze the biosynthesis of trehalose are strongly regulated by insulin signaling via the FoxO transcription factor DAF-16 in the dauer larvae^31^. It was shown that osmotic stress induces DAF-16 dependent trehalose elevation in adult *C. elegans*^29^. Thus, DAF-16 might be a key regulator for trehalose elevation observed in dauer larvae upon ChaP and OsP treatment. In addition to trehalose elevation, ChaP and OsP also induces similar water loss in dauer larvae indicating most of the bulk water (intracellular and extracellular) is lost in similar fashion in both approaches.

Despite similarities in response to ChaP and OsP, we observed one pronounced difference: OsP leads to accumulation of glycerol. Interestingly, biosynthesis of glycerol similar to trehalose also occurs via the glyoxylate shunt and gluconeogenesis. The diverging point should be glyceraldehyde-3-phosphate (Fig. 4A). This provides the dauer larvae with an additional advantage to ChaP, namely the ability to survive freezing (Fig. 4E). Ice crystal formation is one of the factors that results in lethality upon freezing. Several strategies have been proposed to prevent the damage generated due to freezing, one of them is accumulation of polyhydroxy alcohols^32^. With 20% of body water (Fig. 1B), glycerol accumulated upon osmotic preconditioning confers freezing tolerance by preventing ice crystal formation. The synthesis of glycerol from glyceraldehyde-3-phosphate is catalysed by GPDH-1 and GDPH-2^33^, the expression of these enzymes should be differently regulated upon OsP and ChaP. The dissimilarities in response to ChaP and OsP might be due to differences in subset of neurons that modulate the water loss response. TRP channels that mediate hygrosensation^34^ (changes in the humidity levels) might be specifically activated in response to ChaP, whereas tyraminergic neurons might mediate the response to OsP^35^. Future studies could address how these neurons activate differential biochemical response in ChaP and OsP.

OsP provided us a rare opportunity to demonstrate that the metabolic rate of an organism is significantly reduced while the anhydrobiotic program is initiated. As OsP occurs in fluid medium, it had a technical advantage over the conventional ChaP approach for measuring caloric output. It was hypothesized that the anhydrobiotic capacity of the dauer larvae is attributable to their hypometabolic state^36^. Under normal conditions, it would be extremely challenging for an organism to reduce its metabolic rate from an active state to undetectable levels. Dauer larvae in hypometabolic state possess an advantage, as it is thermodynamically more feasible to reduce their metabolism from hypometabolic to undetectable level. For the first time, these microcalorimetry experiments demonstrate the ability of the dauer larvae to transit from a non-preconditioned to anhydrobiotic state. This process is aided by a significant reduction in metabolic rate to promote survival during extreme conditions. Future studies could address how dauer metabolic rate is regulated by the central regulators, such as insulin, steroid hormone, and AMP-activated protein kinase signaling^37^. These pathways are known to reduce the metabolic rate and the transition from TCA cycle to gluconeogenesis at basal dauer state and thus may also modulate metabolism during preconditioning.

In summary, we report that *C. elegans* dauer larvae possess a program that is activated by osmotic pressure or mild desiccation enabling dauer larvae to survive various kinds of abiotic stress. Further understanding of how this program operates at a mechanistic level can provide insights into engineering novel preservative techniques for cells and tissues.

## Methods

### Materials and *C. elegans* strains

[1-^14^C]-acetate (sodium salt) was purchased from Hartmann Analytic (Braunschweig, Germany). All other chemicals were purchased from Sigma-Aldrich (Taufkirchen, Germany). The Caenorhabditis Genetic Centre (CGC) provided the *C. elegans* strain wild type (N2), *daf-2*(*e1370*) and the *E. coli* strain NA22. The compound mutant strains of *daf-2*(*e1370*)*III;lea-1*(*tag1676*)*V, tps-2*(*ok526*)*II;daf-2*(*e1370*)*III;tps-1*(*ok373*)*X*(*daf-2;ΔΔtps*), *daf-2*(*e1370*)*III;icl-1*(*ok531*)*V* were generated during this or previous studies as published^18^^,^^38^.

### Osmotic preconditioning and desiccation survival assay

For osmotic preconditioning, dauer larvae were collected from complete S basal medium or NGM agar plates in 15 ml tubes. They were washed for at least 3–4 times with water to remove all the debris and distributed into 15 ml tubes. 7.5 ml of freshly prepared 0.3 M PEG1000 was added to dauer pellet and incubated on a horizontal shaker at 25 °C. After 18 h of incubation, dauers were centrifuged at 800*g* for 3 min and the supernatant was removed. 7.5 ml of 0.6 M PEG1000 was added to the dauer pellet and incubated on a horizontal shaker at 25 °C for 2–3 h. Dauer larvae treated with water were used as a control for the experiments. In order to remove any reminiscent PEG1000 adhered to the dauers, they were transferred to Isopore TETP membranes (8 μm pore size, Millipore, USA) washed under suction at least 5–6 times with water. Chamber preconditioning was performed according to previous protocol^17^. After osmotic and chamber preconditioning the dishes were transferred to harsh desiccation (60% RH) chamber for 1 day. After one day of harsh desiccation they were rehydrated with 500 μl of distilled water for 2–3 h. They were transferred to agar plates with food and kept at 15 °C for overnight. The following day, survivors and total number of worms in each plate were counted. Survival rate of each condition was calculated as the percentage of survivors in that plate. All the survival assays were performed on different days with at least two technical replicates.

### Water loss and trehalose measurement

Water loss and trehalose measurements were performed according to previous report^17^.

### Isothermal microcalorimetry measurements

To measure the heat production of dauer larvae during osmotic preconditioning with PEG1000, *daf-2* dauers were collected from liquid culture and washed 3–4 times to remove bacteria and debris. After 18 h incubation in 0.3 M PEG1000, dauers were centrifuged at 800*g* for 3 min and the supernatant was removed and 0.6 M PEG1000 was added. Around 2,500 dauers in a volume of 200 μl per each condition were pipetted into a 4 ml glass ampoule (TA Instruments, New Castle, DE, USA) and these ampoules were then sealed with aluminum caps equipped with sealing discs (TA Instruments, New Castle, DE, USA). Isothermal calorimetric measurements were performed with a TAMIII (Thermal Activity Monitor) instrument (Waters GmbH, Eschborn, Germany) equipped with 12 microcalorimeters in twin configuration (one side for the sample the other for a steel reference) to continuously monitor the metabolic heat produced by dauers at 25 °C for up to 120–200 min. The samples were held in the TAM III in a waiting position for 15 min before complete insertion followed by 45 min equilibration. Thermograms were recorded at least in three biological replicates with four technical replicates. Mean metabolic rate was calculated as an average of the first 20 min of measurement and it represents the summation of heat flow of approximately 2,500 dauers per condition.

### Radiolabeling of *C. elegans* dauer, metabolite extraction and 2D-TLC

The above-mentioned procedures were performed according to previous report^19^.

### Measurement of glycerol in lysates and its identification from TLC plate

Glycerol measurements were performed using Glycerol assay kit (Sigma-Aldrich Chemie GmbH, Germany). The dauer samples per condition were immediately collected in 1 ml distilled water, flash frozen in liquid nitrogen, and stored at − 80 °C. They were homogenized by freezing in liquid nitrogen and subsequent thawing in a sonication bath, this cycle of freeze thawing was repeated 5 times. The debris was pelleted by centrifugation at 25,000*g* for 1 min at 4 °C. 30 μl of the supernatant was used for determining soluble protein amounts using a micro BCA assay kit (Thermo Fisher Scientific, Germany). All the reagents of the Glycerol assay kit except the enzyme mix were brought to room temperature. The protocol was optimized for dauer lysate by diluting them to 1:10 and incubating the plates for 25 min. Glycerol concentration in the lysates was calculated based on the absorbance (570 nm) in the samples and normalized to soluble protein amounts.

Aqueous fractions from the osmotic preconditioned sample was separated by high performance thin layer chromatography (HPTLC), using 1-propanol-methanol-ammonia (32%)-water (28:8:7:7 v/v/v/v) as first, dried for 15 min and 1-butanol-acetone-glacial acetic acid–water (35:35:7:23 v/v/v/v) second dimension respectively. Glycerol standard was run on both dimensions, only the region where the standards run was stained with 0.5% potassium permanganate in 1 N sodium hydroxide^39^ and the intersection region was scraped out from the TLC plate. The scraped-out silica was extracted with 10 ml of 50% methanol twice. The fractions were combined, dried under vacuum and dissolved in 100 μl of water.

For derivatization, 850 μl of 4 N sodium hydroxide, 500 μl of hexane, 100 μl of benzoyl chloride was added to 100 μl concentrated fraction in a 10 ml glass tube. The mixture was incubated at 40 °C for 4 h. 1 ml of water was added to this mixture and centrifuged at 2,500*g* for 2 min. The upper phase (organic phase) was transferred to a glass tube, dried under vacuum and dissolved in 100 μl water. The upper phase (organic phase) was filtered through the PTFE filter (GE healthcare) to remove particle debris. The filtered organic phase was subjected to LC–MS for separation purposes. 20 μl of the filtered organic phase was separated on Synergi 2.5 µm Fusion-RP 100 Ȧ LC Column 100 × 2 mm (Phenomenex). The mobile phase was composed of 0.01% Formic acid in water (eluent A) and 0.01% Formic acid in acetonitrile (eluent B). The following gradient program was used. Eluent B: from 50 to 100% within 12 min; 100% from 12 to 17 min; equilibration of 50% from 17 to 25 min. The flow rate was set at 0.3 ml/min. Mass spectrometry detection was carried out using G2-S QTof with electro spray ionization (ESI). MSe mode was used to detect the analytes in positive ionization mode. The spray voltage was set at 300 V and the source temperature was operated at 120 °C. Desolvation gas flow was set to 800 l/h with 450 °C. Sodium adduct of glycerol was detected in positive mode. Exact monoisotopic mass was 427.11521 and mass error in parts per million (ppm) was 4.0.

### Freezing survival assay

Dauer larvae were collected from liquid culture and washed few times with water to remove debris and then resuspended in water. Non-preconditioned and osmotic preconditioned dauers were transferred to Isopore TETP membranes (8 μm pore size, Millipore, USA) washed under suction at least 5–6 times (minimum) with water. The membrane was immediately transferred to a 35 mm plastic petri dish, the lid was closed, wrapped with parafilm and transferred to − 80 °C. Several replicates were prepared per each condition at the beginning of the experiment and for each experimental time point two technical replicates were thawed. At an experimental time point the petri dishes were removed from − 80 °C, thawed for 5–10 min at room temperature and 500 μl of water was added to allow the worms to rehydrate for 2–3 h. Rehydrated dauers were transferred to NGM agar plates with food and allowed to recover. Survival rate of each condition was calculated as the percentage of survivors in that plate. All the survival assays were performed on different days with at least two technical replicates.

### Detection of LEA-1 and generation of *daf-2; lea-1*

For detection of proteome changes, including upregulation of LEA-1 during preconditioning, 2D-DIGE was performed as previously described in^19^. *daf-2;lea-1* strain was generated in the following way. Fifteen μl of master mix (AAATGAGAAGCCGATTGCGG) crRNA-IDT-sgl (5 μM), (CTGATAGTAAATATAGTTGG) crRNA-IDT-sg6 (5 μM), CAS9-Protein NLS (12.5 μM), tracerRNA-IDT (12.5 μM), dpy10 Oligo-IDT (733 nM), dpy-10-sgRNA-IDT (2.5 μM), 5 μl protein buffer stock (3 ×) was injected in young adults of N2 strain. Progeny of the rescued strains were genotyped for homozygous deletion with PCR primers for three generations. One of the several lines obtained was randomly selected and outcrossed twice with wild type strain. The outcrossed strain was crossed with *daf-2*(*e1370*) strain, *daf-2*(*e1370*) homozygosity was confirmed by 100% dauer formation and *lea-1*(*tag1676*) deletion was confirmed by PCR genotyping.

## Supplementary information

Supplementary Information 1.

Supplementary Information 2.
