# Supplementary material for: C. elegans possess a general program to enter cryptobiosis that allows dauer larvae to survive different kinds of abiotic stress
Source: Sci Rep. 2020 Aug 10;10:13466. doi: 10.1038/s41598-020-70311-8 (PMC7417548; doi:10.1038/s41598-020-70311-8)
Supplement: Supplementary file 1 — Supplementary Information 1. [file 41598_2020_70311_MOESM1_ESM.pdf]

Supplementary Information

C. elegans possess a general program to enter cryptobiosis that allows dauer larvae to survive different kinds of abiotic stress

Vamshidhar R. Gade[1], Sofia Traikov[1], Jana Oertel[2], Karim Fahmy[2], Teymuras V. Kurzchalia*[1]

1. Max Planck Institute of Molecular Cell Biology and Genetics, Dresden, Germany.

2. Institute of Resource Ecology at the Helmholtz-Zentrum Dresden-Rossendorf, Dresden, Germany.

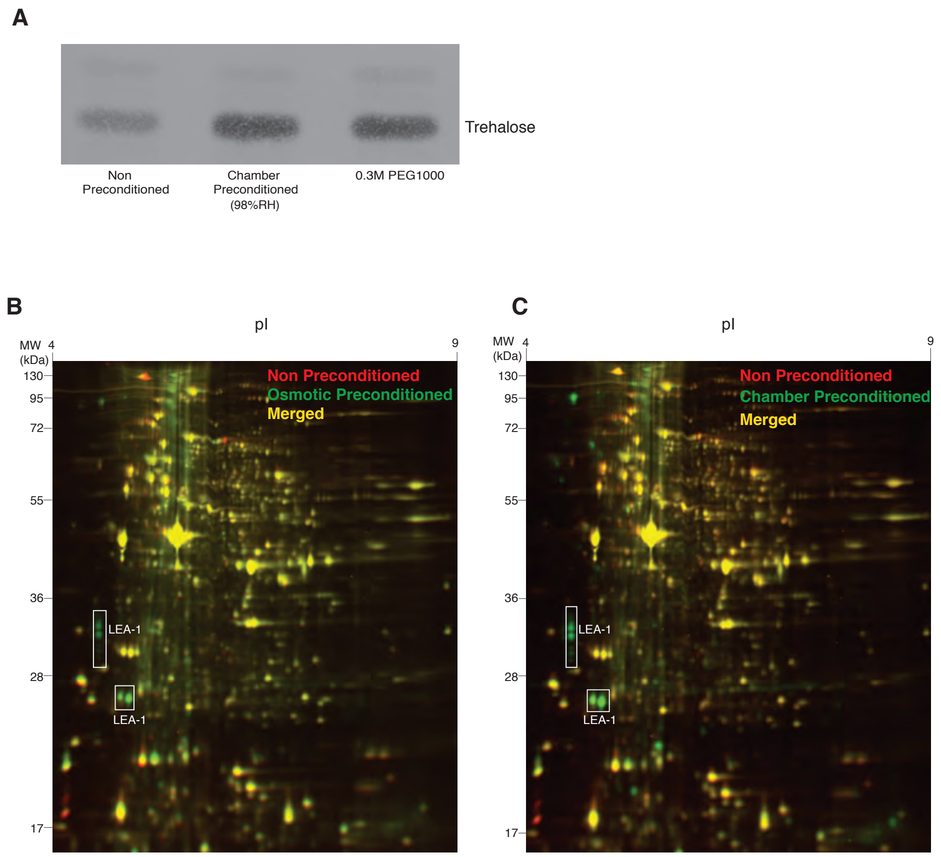
A
Trehalose
Non Preconditioned
Chamber Preconditioned (98%RH)
0.3M PEG1000
B
pI
MW (kDa)
4
9
130
95
72
55
36
28
17
Non Preconditioned
Osmotic Preconditioned
Merged
LEA-1
LEA-1
C
pI
MW (kDa)
4
9
130
95
72
55
36
28
17
Non Preconditioned
Chamber Preconditioned
Merged
LEA-1
LEA-1

Figure S1

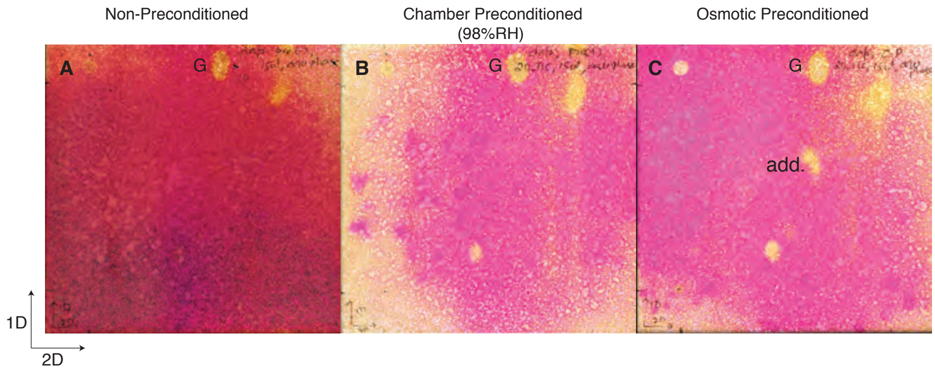
Non-Preconditioned
Chamber Preconditioned
(98%RH)
Osmotic Preconditioned
A
G
B
G
C
G
add.
1D
2D

Figure S2

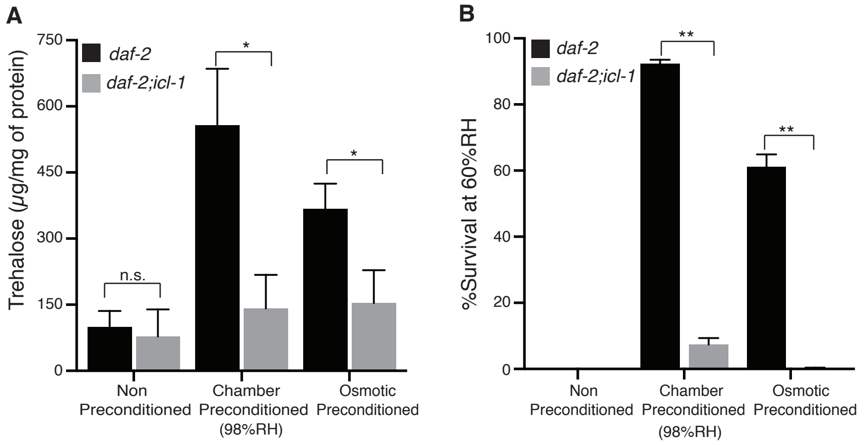
A
Trehalose (μg/mg of protein)
750
600
450
300
150
0
daf-2
daf-2;icl-1
n.s.
*
*
Non
Preconditioned
Chamber
Preconditioned
(98%RH)
Osmotic
Preconditioned
B
%Survival at 60%RH
100
80
60
40
20
0
daf-2
daf-2;icl-1
**
**
Non
Preconditioned
Chamber
Preconditioned
(98%RH)
Osmotic
Preconditioned

Figure S3

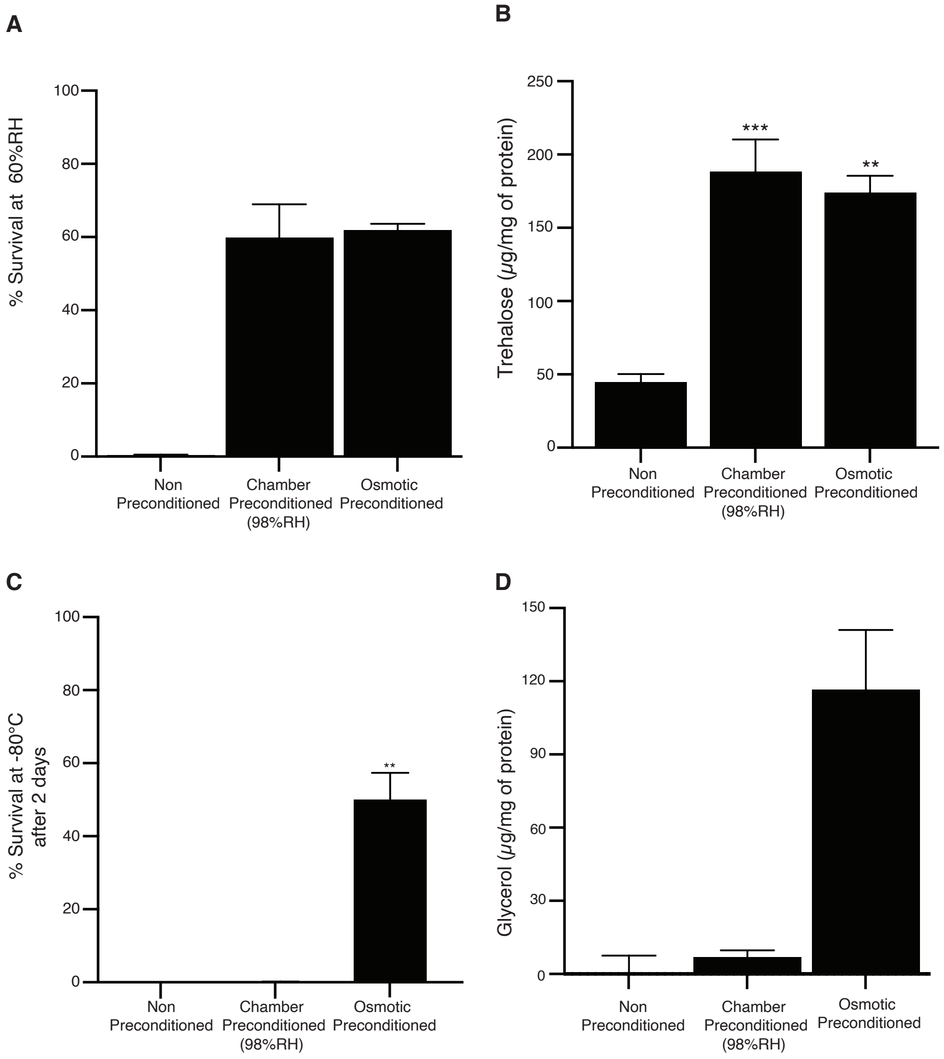
A
% Survival at 60%RH
100
80
60
40
20
0
Non Preconditioned
Chamber Preconditioned (98%RH)
Osmotic Preconditioned
B
Trehalose (μg/mg of protein)
250
200
150
100
50
0
***
**
Non Preconditioned
Chamber Preconditioned (98%RH)
Osmotic Preconditioned
C
% Survival at -80°C after 2 days
100
80
60
40
20
0
**
Non Preconditioned
Chamber Preconditioned (98%RH)
Osmotic Preconditioned
D
Glycerol (μg/mg of protein)
150
120
90
60
30
0
Non Preconditioned
Chamber Preconditioned (98%RH)
Osmotic Preconditioned

Figure S4
