# Supplementary material for: C. elegans possess a general program to enter cryptobiosis that allows dauer larvae to survive different kinds of abiotic stress
Source: Sci Rep. 2020 Aug 10;10:13466. doi: 10.1038/s41598-020-70311-8 (PMC7417548; doi:10.1038/s41598-020-70311-8)
Supplement: Supplementary file 2 — Supplementary Information 2. [file 41598_2020_70311_MOESM2_ESM.docx]

***C. elegans* possess a general program to enter cryptobiosis that allows dauer larvae to survive different kinds of abiotic stress**

Vamshidhar R. Gade^1^, Sofia Traikov^1^, Jana Oertel^2^, Karim Fahmy^2^, Teymuras V. Kurzchalia*^1^

1. Max Planck Institute of Molecular Cell Biology and Genetics, Dresden, Germany.

2. Institute of Resource Ecology at the Helmholtz-Zentrum Dresden-Rossendorf, Dresden, Germany.

Corresponding Author: Teymuras Kurzchalia, PhD

MPI-CBG, Pfotenhauerstrasse 108

01307, Dresden,

Tel: +49 351 210 2567

Fax: +49 351 2101489

Email: [kurzchalia@mpi-cbg.de](mailto:kurzchalia@mpi-cbg.de)

Lead contact: Teymuras Kurzchalia

**Figure Legends**

**Figure S1: Biochemical changes upon osmotic preconditioning in dauers**.

A) Dauer larvae treated with 0.3MPEG1000 upregulates trehalose levels. 1D Thin layer chromatography (TLC) of sugars visualized by molish staining. B&C) LEA-1 is upregulation upon osmotic and chamber preconditioning. Overlay of false-colored 2D-DIGE images comparing dauer proteomes before (red) and after (green) osmotic and chamber preconditioning. The entire proteome is shown.

**Figure S2: Detection of glycerol from the aqueous fraction of osmotic preconditioned sample.**

A-C) 2D-Thin layer chromatography (TLC) of metabolites from dauers that were non-preconditioned, chamber and osmotic preconditioned respectively. Glycerol was visualized with potassium permanganate staining. Enumerated spot add. co-runs with glycerol standard.

**Figure S3: Osmotic preconditioned dauer larvae utilize glyoxylate shunt to survive harsh desiccation.**

A) Glyoxylate shunt is essential for trehalose elevation in osmotic preconditioned dauers. Error bars indicate standard deviation of three independent experiments with three biological replicates. Statistical comparison was done with unpaired t-test with Holm Sidak’s method. *p<0.05. B) Survival rate of *daf-2* and *daf-2;icl-1* osmotic preconditioned dauers to desiccation. Error bars indicate data of two independent experiments with two technical replicates. Statistical comparison was done with unpaired t-test with Holm Sidak’s method. *p<0.05.

**Figure S4: Osmotic preconditioning enhances survival of wild type (*N2*) dauer larvae to harsh desiccation and freezing**.

A) Osmotic preconditioned wild type (N2) dauer larvae survive to harsh desiccation. Error bars indicate standard deviation of two technical replicates. For Water n = 507, Chamber preconditioned n = 340, Osmotic preconditioned n = 1672. B) Trehalose upregulation in wild type (N2) dauer larvae upon osmotic preconditioning. Error bars indicate standard deviation of three biological replicates with three technical replicates. Statistical comparison was performed with one-way ANOVA with Dunnett’s multiple comparisons test. ** p<0.05, *** p<0.001. C) Osmotic preconditioned wild type (N2) dauer larvae survive to freezing. Error bars indicate standard deviation of two biological replicates with two technical replicates. Statistical comparison was performed with one-way ANOVA with Dunnett’s multiple comparisons test. ** p<0.05. For non-preconditioned n = 445, Chamber preconditioned n =1363, Osmotic preconditioned n = 1435. D) Glycerol upregulation in wild type (N2) dauer larvae upon osmotic preconditioning. Error bars indicate standard deviation of three technical replicates.
